# Supplementary material for: Labour pain relief practice by maternal health care providers at a tertiary facility in Kenya: An institution-based descriptive survey
Source: PLoS One. 2024 Mar 7;19(3):e0299211. doi: 10.1371/journal.pone.0299211 (PMC10919675; doi:10.1371/journal.pone.0299211)
Supplement: Attachment — Submitted filename: Reviewed PONE-D-23-09684.docx [file pone.0299211.s002.docx]

PLOS ONE

Labour pain relief practice by maternal health care providers in Kenya: An institutionbased descriptive survey

--Manuscript Draft--

| Manuscript Number: | PONE-D-23-09684 |
| --- | --- |
| Article Type: | Research Article |
| Full Title: | Labour pain relief practice by maternal health care providers in Kenya: An institutionbased descriptive survey |
| Short Title: | Labour pain relief practice by maternal health care providers in Kenya. |
| Corresponding Author: | Kimbley Asaso Omwodo, MD  Moi University College of Health Sciences Eldoret, Uasin Gishu KENYA |
| Keywords: | Pain relief; Labour; practice; Barriers; Kenya. |
| Abstract: | Background: Although pain relief is a crucial component of modern obstetric care, it remains a poorly established service in sub-Saharan countries such as Kenya.  Maternal health care providers have an extensive role to play in meeting the analgesic needs of women during childbirth. This study sought to examine the practice of labour pain relief among Kenyan maternal health care providers.  Methods: This was an institution-based, cross-sectional, descriptive survey. The study included midwives, obstetricians, and anaesthesiologists (n=120) working at the second-largest tertiary facility in Kenya. A structured, self-administered questionnaire was used. The labour pain relief practice, knowledge, attitude, and perceived barriers to labour pain management were described.  Results: One hundred and seventeen respondents participated in the study representing a response rate of 97.5%. More than half of maternal health care providers routinely provided the service of labour pain relief (61.5%). Sixty-four (88.9%) respondents reported providing pharmacological and non-pharmacological methods, while 11.1% provided only pharmacological ones. The most common pharmacological method prescribed was non-opioids (12.8%). The most preferred non-pharmacological method of pain management was touch and massage (93.8%). Regional analgesia was provided by 3.4% of the respondents. More than half of the respondents (53%) had poor knowledge of labour pain relief methods. Almost all (94%) of the respondents had a positive attitude towards providing labour pain relief. Non-availability of drugs and equipment (58.1%), lack of clear protocols and guidelines (56.4%), and absence of adequate skilled personnel (55.6%) were reported as the health system factors that hinder the provision of labour analgesia.  Conclusions: More than half of maternal health care providers routinely relieve labour pain. Epidural analgesia is still relatively underutilized. There is a need to develop institutional labour pain management protocols to meet the analgesic needs of women during childbirth |
| Order of Authors: | Eliazaro Gabriel Ouma |
|  | Omenge Orango |
|  | Edwin Were |
|  | Kimbley Asaso Omwodo |
| Additional Information: |  |
| Question | Response |
| Financial Disclosure  Enter a financial disclosure statement that describes the sources of funding for the work included in this submission. Review the [submission guidelines](http://journals.plos.org/plosone/s/submission-guidelines#loc-financial-disclosure-statement) for detailed | The author(s) received no specific funding for this work. |

| requirements. View published research articles from [PLOS ONE](http://journals.plos.org/plosone/) for specific examples.  This statement is required for submission and will appear in the published article if the submission is accepted. Please make sure it is accurate. |  |
| --- | --- |
| Unfunded studies Enter: The author(s) received no specific funding for this work. Funded studies Enter a statement with the following details: - Initials of the authors who received each award - Grant numbers awarded to each author - The full name of each funder - URL of each funder website - Did the sponsors or funders play any role in the study design, data collection and analysis, decision to publish, or preparation of the manuscript? - NO - Include this sentence at the end of your statement: The funders had no role in study design, data collection and analysis, decision to publish, or preparation of the manuscript. - YES - Specify the role(s) played. |  |
| * typeset |  |
| Competing Interests  Use the instructions below to enter a competing interest statement for this submission. On behalf of all authors, disclose any [competing interests](http://journals.plos.org/plosone/s/competing-interests) that could be perceived to bias this work—acknowledging all financial support and any other relevant financial or nonfinancial competing interests.  This statement is required for submission and will appear in the published article if the submission is accepted. Please make sure it is accurate and that any funding sources listed in your Funding Information later in the submission form are also declared in your Financial Disclosure | The authors have declared that no competing interests exist. |

| statement.  View published research articles from [PLOS ONE](http://journals.plos.org/plosone/) for specific examples. |  |
| --- | --- |
| NO authors have competing interests Enter: The authors have declared that no competing interests exist. Authors with competing interests Enter competing interest details beginning with this statement: I have read the journal's policy and the authors of this manuscript have the following competing interests: [insert competing interests here] |  |
| * typeset |  |
| Ethics Statement  Enter an ethics statement for this submission. This statement is required if the study involved:   - Human participants - Human specimens or tissue - Vertebrate animals or cephalopods - Vertebrate embryos or tissues - Field research   Write "N/A" if the submission does not require an ethics statement.  General guidance is provided below. Consult the [submission guidelines](http://journals.plos.org/plosone/s/submission-guidelines#loc-guidelines-for-specific-study-types) for detailed instructions. Make sure that all information entered here is included in the Methods section of the manuscript. | Ethics approval and consent to participate Ethical clearance was obtained from the Moi University Institutional Research Ethics Committee (IREC)- REF: IREC/2020/144. Permission from MRTH administration was also obtained. Written informed consent was taken from each subject before enrolment in the study following Good Clinical Practice (GCP) principles, and all methods were carried out in accordance with the Declaration of Helsinki. All data were maintained as confidential, and no individual was identified in the dissemination of findings. |

Format for specific study types

Human Subject Research (involving human

participants and/or tissue)

Give the name of the institutional review

board or ethics committee that approved the

study

•

Include the approval number and/or a

statement indicating approval of this

research

•

Indicate the form of consent obtained

(

written/oral) or the reason that consent was

not obtained (e.g. the data were analyzed

anonymously)

•

Animal Research (involving vertebrate

animals, embryos or tissues)

Provide the name of the Institutional Animal

Care and Use Committee (IACUC) or other

relevant ethics board that reviewed the

study protocol, and indicate whether they

approved this research or granted a formal

waiver of ethical approval

•

Include an approval number if one was

obtained

•

If the study involved

non-human primates

,

add

additional details

about animal welfare

and steps taken to ameliorate suffering

•

If anesthesia, euthanasia, or any kind of

animal sacrifice is part of the study, include

briefly which substances and/or methods

were applied

•

Field Research

Include the following details if this study

involves the collection of plant, animal, or

other materials from a natural setting:

Field permit number

•

Name of the institution or relevant body that

granted permission

•

Data Availability

Authors are required to make all data

underlying the findings described fully

available, without restriction, and from the

time of publication. PLOS allows rare

exceptions to address legal and ethical

concerns. See the

[PLOS Data Polic](http://journals.plos.org/plosone/s/data-availability)

[y](http://journals.plos.org/plosone/s/data-availability)

and

[FA](http://journals.plos.org/plosone/s/data-availability#loc-faqs-for-data-policy)

[Q](http://journals.plos.org/plosone/s/data-availability#loc-faqs-for-data-policy)

for detailed information.

No - some restrictions will apply

| A Data Availability Statement describing where the data can be found is required at submission. Your answers to this question constitute the Data Availability Statement and will be published in the article, if accepted. |  |
| --- | --- |
| Important: Stating ‘data available on request from the author’ is not sufficient. If your data are only available upon request, select ‘No’ for the first question and explain your exceptional situation in the text box. |  |
| Do the authors confirm that all data underlying the findings described in their manuscript are fully available without restriction? |  |
| Describe where the data may be found in full sentences. If you are copying our sample text, replace any instances of XXX with the appropriate details.   - If the data are held or will be held in a public repository, include URLs, accession numbers or DOIs. If this information will only be available after acceptance, indicate this by ticking the box below. For example: All XXX files   are available from the XXX database (accession number(s) XXX, XXX.).   - If the data are all contained within the manuscript and/or Supporting Information files, enter the following: All relevant data are within the manuscript and its Supporting Information files. - If neither of these applies but you are able to provide details of access elsewhere, with or without limitations, please do so. For example:   Data cannot be shared publicly because of [XXX]. Data are available from the XXX Institutional Data Access / Ethics Committee (contact via XXX) for researchers who meet the criteria for access to confidential data.  The data underlying the results presented in the study are available from (include the name of the third party | The datasets generated during the current study are not publicly available as they may contain information that could compromise the privacy of research participants; however, the dataset may be made available from the corresponding author upon reasonable request. |

| and contact information or URL).  • This text is appropriate if the data are owned by a third party and authors do not have permission to share the data.  * typeset |  |
| --- | --- |
| Additional data availability information: |  |

Manuscript	[Click here to access/download;Manuscript;Plos one manuscript.docx](https://www.editorialmanager.com/pone/download.aspx?id=32982875&guid=aa6ca53b-62fa-464d-9e94-2d806ca860ea&scheme=1)

# 1 Labour pain relief practice by maternal health care 2 providers in Kenya: An institution-based descriptive

3 **survey.**
4 Gabriel Ouma^1^, Omenge Orango^2^, Edwin Were^3^ and Kimbley Omwodo^4*^

5

6

7

8^1^ Department of Reproductive Health, Moi University, School of Medicine, Eldoret, Kenya.
9 ^2^ Department of Reproductive Health, Moi University, School of Medicine, Eldoret, Kenya. 10	^3^ Department of Reproductive Health, Moi University, School of Medicine, Eldoret, Kenya.

11	^4^ Department of Reproductive Health, Moi University, School of Medicine, Eldoret, Kenya.

12

13 Corresponding author*
14 E-mail : kimbleyomwodo@alumni.harvard.edu

15

16	Conceived and designed the experiments: GO OO EW. Performed the experiments: GO 17	Analyzed the data: GO KO. Wrote the paper: GO KO.

18

**Background:** Although pain relief is a crucial component of modern obstetric care, it remains a poorly established service in sub-Saharan countries such as Kenya. Maternal health care providers have an extensive role to play in meeting the analgesic needs of women during childbirth. This study sought to examine the practice of labour pain relief among Kenyan maternal health care providers.

**Methods:** This was an institution-based, cross-sectional, descriptive survey. The study included midwives, obstetricians, and anaesthesiologists (n=120) working at the secondlargest tertiary facility in Kenya. A structured, self-administered questionnaire was used. The labour pain relief practice, knowledge, attitude, and perceived barriers to labour pain management were described.

**Results:** One hundred and seventeen respondents participated in the study representing a response rate of 97.5%. More than half of maternal health care providers routinely provided the service of labour pain relief (61.5%). Sixty-four (88.9%) respondents reported providing pharmacological and non-pharmacological methods, while 11.1% provided only pharmacological ones. The most common pharmacological method prescribed was nonopioids (12.8%). The most preferred non-pharmacological method of pain management was touch and massage (93.8%). Regional analgesia was provided by 3.4% of the respondents.

More than half of the respondents (53%) had poor knowledge of labour pain relief methods. Almost all (94%) of the respondents had a positive attitude towards providing labour pain relief. Non-availability of drugs and equipment (58.1%), lack of clear protocols and guidelines (56.4%), and absence of adequate skilled personnel (55.6%) were reported as the health system factors that hinder the provision of labour analgesia.

**Conclusions:** More than half of maternal health care providers routinely relieve labour pain. Epidural analgesia is still relatively underutilized. There is a need to develop institutional labour pain management protocols to meet the analgesic needs of women during childbirth.

**Keywords:** Pain relief, labour, practice, barriers, Kenya.

# Background

Labour pain management is a critical consideration in modern obstetrics. Although the childbirth experience is subjective and multifaceted, pain associated with labour has been described as one of the most intense forms of pain [1, 2]. Adverse consequences of labour pain may result from neuroendocrine stress responses, including increasing maternal peripheral vascular resistance and decreasing uteroplacental perfusion [3, 4]. Moreover, unrelieved labour pain has been shown to contribute to the development of postpartum psychological trauma, including postpartum depression[5].

Labour pain relief practice reflects a complex balance between the knowledge, values, and interests of maternal health care providers (MHCPs) and parturients. Both pharmacological and non-pharmacological approaches are necessary for effective labour pain management.

The ideal analgesic should be safe, effective, and based on the parturients' preferences.[6].

In developed economies, pain relief during labour is an integral part of intrapartum care [7], with most studies focusing on comparing the effectiveness of various methods and alternative therapies[8, 9]. However, the practice of labour pain management in Africa is suboptimal, with institutional provision reported to be as low as 13.8% [10].

Limited literature exists on maternal health care provider labour pain practice in Kenya, making it challenging to assess trends in this population. The objective of the study was to assess the labour pain relief practice by maternal healthcare providers working in the second largest public hospital in Kenya.

## Conceptual framework

Interview data were constructed by adopting the Reciprocal Determinism (Reciprocal Causation) model, which has been shown to be superior to other models in assessing pain management practices by healthcare workers [11]. In this study, the internal personal factors included healthcare provider factors, demographic factors (e.g., sex, age, professional cadre, and duration of practice), knowledge and attitude. The environmental factors included the social milieu within which MHCPs continually interact, i.e., health system factors (e.g., availability of adequate skilled personnel, clear protocols and guidelines, drugs and equipment).

# Methods

## Study design

An institution-based, cross-sectional descriptive survey of labour pain relief practice was conducted through a structured, self-administered questionnaire to maternal health care providers.

This study site was the Moi Teaching and Referral Hospital (MTRH), Eldoret, Kenya. MTRH is the second-largest public teaching and referral hospital in Kenya. The Hospital serves mostly residents from the Western Kenya Region (representing at least 22 Counties), parts of Eastern Uganda and Southern Sudan with a population catchment of approximately 24 million. It is also the main teaching centre for Moi University School of Medicine that trains midwives, anesthesiologists and obstetricians with an annual turnover of over 50 practitioners involved directly and indirectly in the management of labour and its outcomes. In the year 2019/2020, an average of 800 women per month gave birth vaginally at MTRH.

Review and approval were obtained from the Moi University School of Medicine/MTRH Institutional Research and Ethics Committee. All participants provided informed consent for data collection.

### Study population

The study population comprised midwives, residents and consultants in both anaesthesia and reproductive health working at MTRH during the data collection period. The study period was between January 1st 2021, and March 31st 2021.

Obstetric caregivers (obstetricians, anesthesiologists, residents and midwives) at MTRH, who are involved in providing labour pain relief and consented to the study, were included.

Medical officer interns, working alongside residents and consultants (who are the primary decision-makers) and with short exposure time in the maternity unit, were excluded from the survey, as they play a secondary role in patient management.

There were no labour pain management guidelines within the maternity unit at the time of the study. The service of labour pain relief was therefore by provider preference.

### Eligibility Criteria

**Inclusion criteria**

- Obstetric caregivers (obstetricians, anesthesiologists, residents and midwives) at MTRH, who are involved in the provision of labour analgesia and consented to the study.

**Exclusion criteria**

- Obstetric caregivers (obstetricians, anesthesiologists, residents and midwives) at MTRH who were on leave and away from the study area during the period of the study.
- First-year residents in both Reproductive Health and Anesthesia who having just joined their respective programs would not have had sufficient exposure time in the facility at the time of the survey.

**Study size**

The study included all the (120) MHCPs of interest at MTRH.

**Selection of participants**

Consecutive sampling method was used until all eligible participants in the study are enrolled. **Study variables**

Dependent variable: labor pain relief practice – choice, pattern and frequency of use of labor analgesia options as self-reported by MHCPs in a standardized questionnaire.

Independent variables: internal personal factors, including MHCPs: Demographic factors (e.g. Sex, Age, Professional cadre and Duration of practice), Knowledge, skills, perception and attitude. Socio-demographic characteristics: age, sex, profession, years of service, department of practice.

The environmental factors including i.e. Patient related factors (e.g., assessed patient knowledge and attitude towards labor analgesia) and the institutional related elements factors (e.g., availability of resources, protocols, guidelines, regulation, and ratio of professionals to patient).

### Study instrument

After obtaining written informed consent, MHCPs who met the inclusion criteria were requested to complete a paper-based structured questionnaire. The questionnaire contained 44 questions and was self-administered. The study adopted a questionnaire based on multiple similar studies per the conceptual framework and study objectives [13, 14].

The questionnaire comprised the following sections:

Section A: sociodemographic characteristics, i.e., sex, age, professional cadre, and duration of practice. Section B assessed the provider’s knowledge and attitude towards labour analgesia. A total of 10 items were included in the questionnaire to assess the respondents’ knowledge, and five items were included to assess the respondents’ attitudes. The reliability analysis of these ten items on knowledge was performed and found to be at an acceptable level of standardized Cronbach’s alpha [α =.718].

Participants’ overall knowledge level was categorized using a modified Bloom’s cut-off point: good if the score was between 80% and 100% (12–15 points), moderate if the score was between 50% and 79% (7–11 points), and poor if the score was less than 50% (˂7 points).

Attitudes towards labour pain relief were assessed using five Likert-type items. The response options for these items were ‘disagree,’ ‘unsure,’ and ‘agree.’ The reliability of these five items was acceptable with a standardized Cronbach’s alpha (α =.804). Similarly, the attitude of healthcare providers towards the provision of labour pain relief was categorized using the original Bloom cut–off point. Attitude was considered positive if the score was 80%–100% (12–15 points), neutral if the score was 60%–79% (9–11 points) and negative if the score was less than 60% (˂ 9 points)[15]. A positive attitude towards the provision of labour pain relief meant perceiving that labour pain is significant enough to warrant intervention and that the provision of labour pain relief should be routine and not an exception.

Section C included questions on the type and frequency of use of the various forms of labour analgesia.

Only participants who responded to be providing any form of labour pain relief’routinely’ were considered to be practising the provision of labour analgesia.

Section D assessed the factors influencing the provision of labour pain relief and employed a 5-point Likert scale from strongly agree to strongly disagree. The first three questions assessed the health system factors influencing the provision of labour analgesia, while the remaining nine assessed other perceived barriers to the provision of labour analgesia.

The concluding section enquired about the provider’s willingness to receive further training on labour analgesia.

To assure the reliability and validity of the data, permission was obtained from the administration and the self-administered questionnaire was pretested on 12 healthcare providers i.e., 10% of the study population. The pilot study was conducted at Jaramogi Oginga

Odinga Teaching and referral hospital (JOOTRH), a level 5 hospital located in Kisumu County. The healthcare providers completed the questionnaire on two separate occasions that were two weeks apart. The period of two weeks was considered long enough for participants to have forgotten their responses but not long enough for a real change to occur in their knowledge, practice or barriers experienced. A discussion ensued to select the best terms for clarity of the questions, accuracy of the knowledge measured, and interpretability.

Participants were not informed of the second administration of the questionnaire on the first occasion. Two sets of responses (i.e., the first and the second administration) were used to measure test-retest reliability. The first administration’s responses assessed construct validity and internal consistency reliability.

Cronbach’s alpha with r=0.7 or greater was considered sufficiently reliable. In the reliability analysis, all questions had alpha scores of 0.7 to 0.9, implying respectable to good reliability.

## Results

Of the 120 maternal health care providers approached, 117 responded, representing a 97.5% response rate. Table 1 demonstrates the sociodemographic characteristics of the participants.

184	**Table 1. Sociodemographic characteristics of maternal healthcare providers.**

| **Sociodemographic characteristics** |  | **Frequency** |  | **Percentage** 186 **(%)** |
| --- | --- | --- | --- | --- |

185

| **Sex** Male | 58 | 49.6 |
| --- | --- | --- |
| Female | 59 | 50.4 |
| **Age (years)**  ≤30 | 7 | 6 |
| 31–40 | 84 | 71.8 |
| > 40 | 26 | 14.5 |
| **Min. – Max.** | **27.0–60.0** |  |
| **Mean ± SD.** | **38.44 ± 7.41** |  |
| **Profession**  Anaesthesiologist | 16 | 13.7 |
| Midwife | 48 | 41 |
| Obstetrician | 53 | 45.3 |
| **Duration of practice (years)**  ≤10 | 78 | 66.7 |
| 11- 20 | 35 | 29.9 |
| >20 | 4 | 3.4 |
| **Min. – Max.** | **2.0–26.0** |  |
| **Mean ± SD.** | **9.54 ± 4.94** |  |

187

188 **The pattern of provision of labour analgesics by maternal health care providers.**
189 Seventy-two respondents (61.5%) routinely provided labour pain relief. Of these, 88.9%
190 reported offering both pharmacological and non-pharmacological methods, while 11.1%
191 provided only pharmacological methods. Slightly more than half of all respondents (54.7%)
192 reported routinely providing non-pharmacological methods of labour analgesia. Non-opioids 193 were the most common pharmacological method prescribed by 13.4% (n=15) of the
194 respondents. Nine (8.7%) participants reported routinely providing opioids. Regional analgesia
195 was routinely prescribed by 3.6% (n=4) of respondents. None of the MHCPs routinely
196 practiced labour pain management by inhalational analgesics (Table 2).

197

198	**Table 2. Maternal healthcare providers’ pattern of provision of labour pain relief.**

| **Pain relief method** | **Registrar**  **Anaesthesiol ogist (n=10)** | **Anaesthe siologist**  **(n=6)** | **Midwife (n=48)** | **Obstetrici an (n=19)** | **Registrar**  **Obstetricia n (n=34)** | **Total (n=117)** |
| --- | --- | --- | --- | --- | --- | --- |
| **Opioids**  Missing | 1 | 2 | 9 | 0 | 2 | 14 |
| Never | 3 (33.3%) | 1 (25.0%) | 4 (10.3%) | 1 (5.3%) | 4 (12.5%) | 13 (12.6%) |
| Occasionally | 5 (55.6%) | 1 (25.0%) | 35 (89.7%) | 12 (63.2%) | 23 (71.9%) | 76 (73.8%) |
| On maternal request | 0 (0.0%) | 0 (0.0%) | 0 (0.0%) | 1 (5.3%) | 4 (12.5%) | 5 (4.9%) |
| Routinely | 1 (11.1%) | 2 (50.0%) | 0 (0.0%) | 5 (26.3%) | 1 (3.1%) | 9 (8.7%) |
| **Non-opioids**  Missing | 1 | 2 | 1 | 0 | 1 | 5 |
| Never | 0 (0.0%) | 1 (25.0%) | 6 (12.8%) | 3 (15.8%) | 8 (24.2%) | 18 (16.1%) |
| Occasionally | 7 (77.8%) | 1 (25.0%) | 38 (80.9%) | 10 (52.6%) | 17 (51.5%) | 73 (65.2%) |
| On maternal request | 0 (0.0%) | 0 (0.0%) | 0 (0.0%) | 1 (5.3%) | 5 (15.2%) | 6 (5.4%) |
| Routinely | 2 (22.2%) | 2 (50.0%) | 3 (6.4%) | 5 (26.3%) | 3 (9.1%) | 15 (13.4%) |
| **Inhalational**  Missing | 0 | 2 | 1 | 0 | 1 | 4 |
| Never | 10 (100.0%) | 3 (75.0%) | 47 (100.0%) | 18 (94.7%) | 33 (100.0%) | 109 (96.5%) |
| Occasionally | 0 (0.0%) | 1 (25.0%) | 0 (0.0%) | 0 (0.0%) | 0 (0.0%) | 1 (0.9%) |
| On maternal request | 0 (0.0%) | 0 (0.0%) | 0 (0.0%) | 1 (5.3%) | 0 (0.0%) | 1 (0.9%) |
| **Regional agent**  Missing | 0 | 1 | 3 | 0 | 1 | 5 |
| Never | 6 (60.0%) | 0 (0.0%) | 38 (84.4%) | 4 (21.1%) | 24 (72.7%) | 72 (64.3%) |
| Occasionally | 3 (30.0%) | 4 (80.0%) | 7 (15.6%) | 10 (52.6%) | 9 (27.3%) | 33 (29.5%) |
| On maternal request | 0 (0.0%) | 0 (0.0%) | 0 (0.0%) | 3 (15.8%) | 0 (0.0%) | 3 (2.7%) |
| Routinely | 1 (10.0%) | 1 (20.0%) | 0 (0.0%) | 2 (10.5%) | 0 (0.0%) | 4 (3.6%) |
| **Nonpharmacol**  **ogical** Missing | 0 | 1 | 0 | 0 | 1 | 2 |
| Never | 1 (10.0%) | 2 (40.0%) | 1 (2.1%) | 1 (5.3%) | 3 (9.1%) | 8 (7.0%) |
| Occasionally | 6 (60.0%) | 2 (40.0%) | 11 (22.9%) | 8 (42.1%) | 12 (36.4%) | 39 (33.9%) |
| On maternal request | 2 (20.0%) | 0 (0.0%) | 0 (0.0%) | 1 (5.3%) | 1 (3.0%) | 4 (3.5%) |

Routinely	1 (10.0%)	1 (20.0%)	36 (75.0%)	9 (47.4%)	17 (51.5%)	64 (54.7%)

**Anaesthesiologists - both registrar and consultant:** Non-opioids as routine labour pain relief was reported by 30.8% (n=4) of the anaesthesiologists. Opioids were provided by 23.1% (n=3) of the respondents within this cadre, while regional analgesics and non-pharmacological methods of pain relief were each provided by 15.4% (n=2) of the respondents. Inhalational analgesics were not routinely provided by any of the anaesthesiologist respondents. No response was obtained from 3 of 16 respondents within the cadre.

**Midwives:** Of the 48 midwife respondents, a majority (75.0%) reported providing nonpharmacological methods for labour pain management. Non-opioids were the most routinely provided pharmacological treatment for labour pain by 6.4%. None of the midwife respondents reported routine provision of opioids or regional and inhalational methods for labour pain relief

**Obstetrician - both registrar and consultant:** Twenty–six (50%) obstetrician respondents reported providing non-pharmacological modes of labour pain management. Non-opioids were the primary pharmacological agents provided by the majority (15.4%) of respondents, and 11.8% (n=6) reported providing opioids routinely. Regional analgesics were provided by 3.8% (n=2) of the respondents, while none of the obstetrician respondents reported providing inhalational agents for labour pain management.

Cumulatively, tramadol was the most routinely provided opioid analgesic by 88.9% (n=8) of the maternal health care providers. Buscopan and paracetamol were the most routinely (66.7%) prescribed non-opioid analgesics. Epidural analgesics were the most preferred regional analgesia by 75% (n=3) of MHCPs. The four most routinely prescribed non-pharmacological methods for labour pain relief were touch and massage (93.8%), deep breathing/patterned breathing (Lamaze techniques) (81.3%), maternal movements and positional changes (81.3%) and social support (reassurance) (79.7%) (Table 3).

**Table3. Maternal healthcare providers’ routine labour pain relief methods.**

| **Agent** | **Frequency** | **%*** |
| --- | --- | --- |
| **Opioids** Tramadol | 8 | 88.9 |
| Morphine | 5 | 55.6 |
| Pethidine | 3 | 33.3 |
| Fentanyl | 3 | 33.3 |
| **Reported provision of any opioid** | **9** | **7.7** |
| **Nonopioids** Buscopan | 10 | 66.7 |
| Panadol | 10 | 66.7 |
| Diclofenac | 2 | 13.3 |
| **Reported provision of any non-opioid** | **15** | **12.8** |
| **Regional**  Epidural | 3 | 75.0 |
| Spinal | 2 | 50.0 |
| **Reported provision of any regional** | **4** | **3.4** |
| **Nonpharmacological** Touch and massage | 60 | 93.8 |
| Deep breathing/patterned breathing (Lamaze) | 52 | 81.3 |
| Maternal movements and positional changes | 52 | 81.3 |
| Social support (Reassurance) | 51 | 79.7 |
| Audio analgesia | 24 | 37.5 |
| Yoga | 3 | 4.7 |
| Intermittent local heat and cold therapy | 1 | 1.6 |
| Acupuncture | 1 | 1.6 |
| **Reported provision of any non- pharmacological** | **64** | **54.7** |

***** Percentages do not add to 100% because some respondents reported providing multiple methods for labour analgesia.

**Factors influencing the provision of labour pain relief**

**Providers’ Knowledge**

Only 5 (4.3%) of the maternal health care providers rated as having good knowledge of labour pain relief practises. All the consultant anaesthesiologists and 52.6% of the consultant

230 obstetricians rated moderately regarding overall knowledge of labour analgesia. The
231 proportion of those rated as having poor knowledge of labour pain relief was higher among
232 registrar obstetricians (70.6%). Based on the composite score of 6.7/15, a majority (53.0%) of
233 maternal health care providers at MTRH had poor knowledge of labour analgesia, as assessed 234	using the modified Blooms cut-off points (Table 4).
235 **Table 4. Maternal healthcare providers’ knowledge of labour pain relief.**

| **Cadre** | **Good** | **Moderate** | **Poor** | **Average score*** | **Score**  **%** |
| --- | --- | --- | --- | --- | --- |
| Anaesthesiologist (n=6) | 0 (0.0%) | 6 (100%) | 0 (0.0%) | **9.5** | **63.3** |
| Resident  anaesthesiologist  (n=10) | 0 (0.0%) | 7 (70.0%) | 3 (30.0%) | **7.4** | **49.3** |
| Midwife (n=48) | 1 (2.1%) | 18 (37.5%) | 29 (60.4%) | **6.2** | **41.3** |
| Obstetrician (n=19) | 3 (15.8%) | 10 (52.6%) | 6 (31.6%) | **7.7** | **51.3** |
| Resident obstetrician (n=34) | 1 (2.9%) | 9 (26.5%) | 24 (70.6%) | **6.1** | **40.7** |
| **TOTAL n=117** | **5 (4.3%)** | **50 (42.7%)** | **62 (53.0%)** | **6.7** | **44.7** |

236 * Maximum score of 15
237 In the self-assessment of previous education concerning labour analgesia, 81.2% (n=95) of
238 the participants had a “yes” response. The reported sources of labour pain relief knowledge 239	were as part of the curriculum in previous education (60.8%), in-service education (52.6%), 240	literature/the internet (39.2%), and colleagues (27.8%) (Table 5).

241	**Table 5. Sources of knowledge on labour pain relief.**

242

243

| **Cadre** | **As part of the**  **curriculum in previous education** | **During inservice**  **education**  **(C.M.E,**  **Seminars etc.)** | **literature / the internet** | 244  **From** 245 **colleagues** |
| --- | --- | --- | --- | --- |
| Anesthesiologist  (n=13)  Midwife (n=38)  Obstetrician (n=46) | 76.9  55.3  60.9 | 69.2  42.1  56.5 | 30.8  34.2  45.7 | 246 7.7  23.7 247  36.9 |
| **Total (n=97)** | **60.8** | **52.6** | **39.2** | **27.8** 248 |

249

250

251 A total of 72.6% (n=85) of MHCPs reported being aware of the universal pain assessment

| (N=16) |  |  |  |  |  |  |
| --- | --- | --- | --- | --- | --- | --- |
| Midwife (N=48) | 31.3 |  | 21.8 | 34.8 | 74.0 | 47.9 |

252 tools; however, only 36.8% used these tools to assess labour pain. Of the respondents reporting
253 the use of the universal pain assessment tools, a majority (71.4%) of the anesthesiologist and
254 obstetricians (38.4%) preferred using the visual component, whereas the majority (74.0%) of
255 the midwives preferred the verbal component (Table 6). Notably, 65.8% of respondents were
256 aware of the WHO analgesic ladder. Of these, 47.0% used this tool during labour pain
257 management. Overall, anaesthesiologists had better knowledge of the pain assessment tools 258 than the other cadres surveyed.

259	**Table 6. Maternal healthcare providers’ use of pain assessment tools in labour pain relief.**

**Universal pain assessment tools**

| **Cadre** | **WHO**  **analgesic ladder(n=55)** |  | **Numerical** | **Visual** | **Verbal** | **Total using**  **UPA*** |
| --- | --- | --- | --- | --- | --- | --- |

Anaesthesiologist	81.3	42.9	71.4	28.6

Obstetrician (N=53)	50.9	30.1	30.8	24.5

38.4

|  | **Total† 27.9 41.9** | **53.5** |
| --- | --- | --- |
| **Total (N=117)** | **47.0** | **36.8** |

† Values do not add up to 100% because some respondents reported using more than one tool

**UPA*:** Universal pain assessment tools

There was poor overall knowledge of opioid dose properties, with only 23.7% (n=27) of all the respondents aware that opioids do not have a ceiling effect. More than half (58.1%) of the MHCPs were aware that non-pharmacological pain relief methods are safer than pharmacological analgesics, and 76.1% were also aware that pharmacological pain relief methods increase women’s comfort in labour compared to non-pharmacological analgesics.

**Attitude**

Based on the composite score of 13.3, 88.7% of MHCPs generally had a positive attitude towards the provision of labour analgesia, as assessed using the original Bloom cut-off points (Table 7).

**Table 7. Maternal healthcare providers’ attitude towards labour pain relief.**

| **Cadre** | **Positive** | **Neutral** | **Negative** | **Avg Score†** | **Score %** |
| --- | --- | --- | --- | --- | --- |
| Anaesthesiologist (N=6) | 6(100.0%) | 0(0.0%) | 0(0.0%) | 13.2 | **88** |
| Resident anaesthesiologist (N=10) | 9(90.0%) | 1(10.0%) | 0(0.0%) | 13 | **86.7** |
| Midwife (N=48) | 45(93.8%) | 3(6.3%) | 0(0.0%) | 13.2 | **89.3** |
| Obstetrician (N=19) | 18(94.7%) | 1(5.3%) | 0(0.0%) | 13.4 | **89.5** |
| Resident obstetrician (N=33) | 31(93.9%) | 2(6.1%) | 0(0.0%) | 13.5 | **90** |
| **TOTAL N=116** | **109(94.0%**  **)** | **7(6.0%)** | **0(0.0%)** | **13.3** | **88.7** |

**†**Maximum score of 15

Forty-three (36.8%) respondents expected women to feel pain during labour. A majority (82.1%) of the respondents agreed that labour pain should be relieved, with an equal number also agreeing that relief of labour pain improves the overall maternal experience. However, ten (8.5%) of the study subjects believed that labour is a natural process that does not require any analgesia; 17.1% were unsure, while the remaining 74.4% disagreed (Table 8).

**Table 8. Maternal healthcare providers’ specific attitude toward provision of labour pain relief.**

|  | **Percentage of maternal health care providers’ who agree that:** | | | | **Percentage of maternal health care providers’ who disagree that:** | |
| --- | --- | --- | --- | --- | --- | --- |
| **Cadre**  **Anesthesiologist**  **(N=16)** | Women are  expected to feel pain  during labour | Pain in labour  should be relieved | Relief of  Labour pain  improves  the overall maternal experience | Labour is a natural  process that  does not require analgesia | | Patients complaining of pain  during  labour may be seeking attention |
|  | 18.8 | 100 | 87.5 | 93.8 | | 100 |
| **Midwife (N=48)** | 50 | 66.7 | 70.8 | 50 | | 77.1 |
| **Obstetrician (N=53)** | 30.2 | 90.6 | 90.6 | 90.6 | | 86.8 |
| **Total (N=117)** | 36.8 | 82.1 | 82.1 | 74.4 | | 84.6 |

**Health system factors.**

A majority (91.7%) of maternal health care providers at MTRH reported experiencing health system factors that hindered their provision of labour analgesia. These included the nonavailability of drugs and equipment (58.1%), a lack of clear protocols and guidelines (56.4%) and an absence of adequate skilled personnel (55.6%).

Other barriers/factors hindering the provision of labour pain relief as reported by maternal health care providers at MTRH included the following (N=117):

i.	Fear of foetal distress (47.1%) ii.	Fear of adverse maternal effects (41.8%) iii.	Cost implications (perceived as expensive) (36.7%)

iv.	Fear that it may increase the incidence of caesarean sections and instrumental delivery (34.2%)

Thirteen (11.1%) respondents reported that oftentimes, patients decline labour analgesia.

Almost all the participants (94%) reported that the introduction of labour pain relief guidelines would improve the management of labour at MTRH, while 95.7% indicated that regular courses on effective labour pain relief would be useful in their practice of labour analgesia.

In the bivariate logistic regression analysis, there was no significant association between the provision of labour pain relief and the age, knowledge, and attitude of the MHCPs.

## Discussion

Slightly over half of MHCPs provided labour pain relief routinely (mainly nonpharmacological). This proportion may be considered inadequate considering the hospitals tertiary status and might allude to a lesser provision of labour pain relief in lower-tier facilities in Kenya. However, this proportion was higher than similar studies conducted in Ibadan, Nigeria and Hawassa, Ethiopia [10, 16]. This difference might be due to the inclusion of different-tier public healthcare institutions in the prior studies and, consequently, a difference in the knowledge and availability of resources.

Non-pharmacological labour pain relief methods, such as massage, breathing techniques, maternal movements, positional changes, support, and companionship, were the most commonly used, likely due to their safety profile. This approach is often the first-line option for labour pain management in many settings.

Non-opioid pharmacological methods, particularly Buscopan and paracetamol, were the most commonly prescribed by maternal health care providers. Opioid use was low, potentially due to poor pain scoring, concerns about side effects, and a reliance on non-pharmacological pain relief methods.

While pethidine injection was previously the most prescribed opioid for labour pain relief [17], tramadol was the most preferred opioid reported in this study, as pethidine is now a highly restricted drug at the study facility.

The World Health Organization (WHO) recommends epidural analgesia for healthy pregnant women requesting pain relief during labour, depending on a woman’s preferences [18]. Data on epidurals in developing countries are scarce, but there is a generally low provision of labour epidurals [19, 20]. In this study, only 3.6% of the respondents reported offering regional analgesia routinely. Epidural analgesia is provided to approximately 30% and 73% of women in labour in the United Kingdom and the US, respectively, with increasing rates expected globally [21, 22]. In our setting, epidural analgesia is reserved for women with pregnancies complicated by medical conditions.

MHCPs’ at MTRH had generally poor knowledge of labour pain relief methods, which may hinder accurate assessment, identification, recommendation, and consultation for proper pain relief. These findings were slightly higher than those reported from the Amhara region, Ethiopia, 48.5% [23], but lower than those reported from Ibadan, Nigeria, 66.7% [16]. The difference may be due to the study participant’s variance in demographic characteristics. The study also found that most resident obstetricians (70.6%) and midwives (60.4%) had poor knowledge scores, highlighting potential gaps in reproductive health course content for health professionals.

In the current findings, almost all MHCPs were positive about providing labour pain relief. This is higher than similar studies in Ethiopia, 57.2% [23], which may signify a cultural disparity between the study populations.

In contrast to studies from Ethiopia and Nigeria [23, 24], this study found no significant association between the provision of labour pain relief and the knowledge or attitude of maternal health care providers.

Health system barriers such as non-availability of drugs and equipment, lack of clear protocols and guidelines, and inadequate skilled personnel were reported as significant hindrances (by 91.7%) to the use of labour pain relief methods in this study.

This finding was consistent with studies from Ethiopia [25], Saudi Arabia [26] and Nigeria[19]. **Limitations of the study**

This was a tertiary institution-based study conducted in Eldoret, Kenya; hence, the conclusions can only be generalized to similar-tier hospitals with equal capacity. We also recommend further studies to explore a broader scope of MHCP perspectives to comprehensively address the cause-and-effect relationship of the factors affecting the provision of labour analgesia.

## Conclusions

Maternal health care providers commonly use non-pharmacological methods, such as massage, breathing techniques, and position changes, to relieve labour pain. Although epidural analgesia is the preferred method, it is underused, and most providers have inadequate knowledge about it. Improving education in labour pain management for healthcare professionals and establishing standard procedures in healthcare institutions could help address this knowledge gap

Health system barriers impede the effective use of labour pain management methods. Interventions aimed at addressing these barriers are necessary for improving the quality of care for women during labour and delivery in Kenya. We recommend further studies to explore the in-depth perspectives of providers and pregnant women on labour pain management, as they play a key role in labour and birth.

**Abbreviations**

MTRH Moi Teaching and Referral Hospital

RMBH Riley Mother and Baby Hospital

KAP	Knowledge, attitude and practices

**Declarations:**

**Ethics approval and consent to participate**

Ethics approval and consent to participate Ethical clearance was obtained from the Moi University Institutional Research Ethics Committee (IREC) REF: IREC/2020/144. Permission from MRTH administration was also obtained. Written informed consent was taken from each subject before enrolment in the study following Good Clinical Practice (GCP) principles, and all methods were carried out in accordance with the Declaration of Helsinki. All data were maintained as confidential, and no individual was identified in the dissemination of findings.

**Financial Disclosure statement**

The authors received no specific funding for this work.

**Acknowledgements**

We appreciate the effort of the doctors, anaesthesiologists, and midwives at the Reproductive Health Department of MTRH for their enormous support in participating in the study.

## References

1. Ranta P, Spalding M, Kangas-Saarela T, Jokela R, Hollmén A, Jouppila P, et al. Maternal expectations and experiences of labour pain--options of 1091 Finnish parturients. Acta Anaesthesiol Scand. 1995;39(1):60-6.
2. Melzack R. The myth of painless childbirth (the John J. Bonica lecture). Pain. 1984;19(4):321-37.
3. Brownridge P. The nature and consequences of childbirth pain. Eur J Obstet Gynecol Reprod Biol. 1995;59 Suppl:S9-15.
4. Shnider SM, Wright RG, Levinson G, Roizen MF, Wallis KL, Rolbin SH, et al. Uterine blood flow and plasma norepinephrine changes during maternal stress in the pregnant ewe. Anesthesiology. 1979;50(6):524-7.
5. Hiltunen P, Raudaskoski T, Ebeling H, Moilanen I. Does pain relief during delivery decrease the risk of postnatal depression? Acta Obstet Gynecol Scand.

2004;83(3):257-61.

6. Care PEP. Essential Antenatal, Perinatal and Postpartum Care. Training module WHO. 2002.
7. Nunes VD, Gholitabar M, Sims JM, Bewley S. Intrapartum care of healthy women and their babies: summary of updated NICE guidance. BMJ. 2014;349.
8. Czech I, Fuchs P, Fuchs A, Lorek M, Tobolska-Lorek D, Drosdzol-Cop A, et al. Pharmacological and non-pharmacological methods of labour pain relief— establishment of effectiveness and comparison. International journal of environmental research and public health. 2018;15(12):2792.
9. Labor S, Maguire S. The pain of labour. Reviews in pain. 2008;2(2):15-9.
10. Wakgari N, Mekonnen M, Lema B, Negasu A, Lulu B, Abebe E. Labour pain management practices among obstetric care providers in Hawassa city, Ethiopia. African Journal of Midwifery and Women’s Health. 2020;14(2):1-12.
11. Alzghoul BI, Chew Abdullah NA. Psychosocial theories and pain management practices: A review of empirical research. Mediterranean Journal of Social Sciences. 2015;6(6 S2):60-7.
12. Bandura A. Social cognitive theory of personality. Handbook of personality. 1999;2:154-96.
13. Mulugeta H. The practice of labor analgesia and its perceived Barriers among Health Care providers working in Public Hospitals of Addis Ababa, Ethiopia: Addis Ababa University; 2016.
14. Endalew NS, Tawuye HY, Melesse DY. Knowledge and attitude towards pain relief in labor among final year midwifery students: A cross-sectional study. International Journal of Surgery Open. 2020;24:38-42.
15. Seid MA, Hussen MS. Knowledge and attitude towards antimicrobial resistance among final year undergraduate paramedical students at University of Gondar, Ethiopia. BMC infectious diseases. 2018;18(1):1-8.
16. Ohaeri B, Owolabi G, Ingwu J. Skilled health attendants’ knowledge and practice of pain management during labour in health care facilities in Ibadan, Nigeria. European Journal of Midwifery. 2019;3.
17. Bricker L, Lavender T. Parenteral opioids for labor pain relief: a systematic review. American journal of obstetrics and gynecology. 2002;186(5):S94-S109.
18. WHO. Defining competent maternal and newborn health professionals: background document to the 2018 joint statement by WHO, UNFPA, UNICEF, ICM, ICN, FIGO and IPA: definition of skilled health personnel providing care during childbirth. 2018.
19. Ogboli-Nwasor E, Adaji S, Bature S, Shittu O. Pain relief in labor: a survey of awareness, attitude, and practice of health care providers in Zaria, Nigeria. Journal of pain research. 2011;4:227.
20. Geltore TE, Taye A, Kelbore AG. Utilization of obstetric analgesia in labor pain management and associated factors among obstetric caregivers in public health facilities of Kembata Tembaro Zone, Southern Ethiopia. Journal of Pain Research. 2018;11:3089.
21. NHS patient survey programme. 2019 survey of women’s experience of maternity care. [Internet]. 2019 [cited January 7th 2022]. Available from: [https://www.cqc.org.uk/sites/default/files/20200128_mat19_statisticalrelease.pdf.](https://www.cqc.org.uk/sites/default/files/20200128_mat19_statisticalrelease.pdf)
22. Butwick AJ, Bentley J, Wong CA, Snowden JM, Sun E, Guo N. United States statelevel variation in the use of neuraxial analgesia during labor for pregnant women. JAMA network open. 2018;1(8):e186567-e.
23. Bishaw KA, Sendo EG, Abebe WS. Knowledge, and use of labour pain relief methods and associated factors among obstetric caregivers at public health centers of East Gojjam zone, Amhara region, Ethiopia: a facility based cross-sectional study. BMC pregnancy and childbirth. 2020;20(1):1-9.
24. Almushait M, Ghani RA. Perception toward non-pharmacological strategies in relieving labor pain: an analytical descriptive study. J Nat Sci Res. 2014;4(2).
25. Sahile E, Yemaneh Y, Alehegn A, Nigussie W, Salahuddin M, Yekoye A, et al. Practice of Labour Pain Management Methods and Associated Factors among Skilled Attendants Working at General Hospitals in Tigray Region, North Ethiopia: Hospital Based Cross-Sectional Study Design. Health Science Journal. 2017;11(4).
26. Aschenbrenner AP, Hanson L, Johnson TS, Kelber ST. Nurses’ own birth experiences influence labor support attitudes and behaviors. Journal of Obstetric, Gynecologic & Neonatal Nursing. 2016;45(4):491-501.
