# Supplementary material for: Labour pain relief practice by maternal health care providers at a tertiary facility in Kenya: An institution-based descriptive survey
Source: PLoS One. 2024 Mar 7;19(3):e0299211. doi: 10.1371/journal.pone.0299211 (PMC10919675; doi:10.1371/journal.pone.0299211)
Supplement: Attachment — Submitted filename: Response to Reviewers.docx [file pone.0299211.s003.docx]

Kimbley .A. Omwodo

Moi University

School of Medicine

P.O Box 4606 -30100,

Eldoret- Kenya

Editor-in-Chief

PLOS ONE Journal

Dear Editor-in-Chief,

**Re: Response to Review of Manuscript ID PONE-D-23-09684**

I would like to thank the reviewers for their insightful comments on our manuscript titled "Labour Pain Relief Practice by Maternal Health Care Providers in Kenya: An Institution-Based Descriptive Survey." We appreciate their time and effort in reviewing our work, and we have carefully considered their comments and suggestions for improvement.

Reviewer #1:

Title Concerns: We appreciate the reviewer's concern about the title's scope. We acknowledge that the study was conducted at a single institution, but the intention was to shed light on the labor pain relief practices in this tertiary facility, which often serves as a reference point for medical care in the region. We understand the concern about generalizing to all of Kenya and agree that it may be misleading. Therefore, we have revised the title to "Labour Pain Relief Practice by Maternal Health Care Providers at a Tertiary Facility in Kenya: An Institution-Based Descriptive Survey" to more accurately reflect the study's scope.

Ethical Clearance and Informed Consent: We thank the reviewer for pointing out the need for additional information regarding the ethical clearance and informed consent process. We have updated the manuscript to include the Moi University School of Medicine/MTRH Institutional Research and Ethics Committee's ethical clearance date and number. Additionally, we have clarified that written informed consent was obtained from all participants.

Sample Size and Selection: The reviewer rightly questioned the methodology for sample size determination and participant selection. We have added a brief explanation regarding this. ”The study employed a census that included all the 120 Health care providers of interest at MTRH”. As for participant selection, we used of consecutive sampling to enroll all eligible participants in the study.

Reviewer #2:

The reviewer pointed out an important question about the equal sex distribution of respondents in the methodology. It was coincidental that there was an equal number of male and female participants as the study included all maternal health care providers at the unit.

On sharing a de-identified data set: We are fully committed to promoting transparency and facilitating data sharing in the scientific community. The minimal anonymized data set necessary to replicate our study findings has been made available as Supporting Information files with this submission.

Regarding the ethics statement placement: We appreciate the reviewer's feedback and will ensure that the ethics statement is correctly positioned in the Methods section of our manuscript. We will make the necessary adjustment and delete any redundant appearances in other sections, as requested.

We hope that these revisions address the concerns raised by the reviewers and improve the overall quality and clarity of our manuscript. We would like to express our gratitude to the reviewers for their feedback, which has undoubtedly strengthened our research. We look forward to hearing your decision on the revised manuscript.

Sincerely,

Kimbley Asaso Omwodo.
